# Oncogene-targeting nanoprobes for early imaging detection of tumor

**DOI:** 10.1186/s12951-023-01943-x

**Published:** 2023-06-21

**Authors:** Wenyue Li, Peisen Zhang, Chuang Liu, Yuping Xu, Zhihua Gan, Lei Kang, Yi Hou

**Affiliations:** 1 College of Materials Science and Engineering, College of Life Science and Technology, Beijing University of Chemical Technology, Beijing, 10029 China; 2 Department of Nuclear Medicine, Peking University First Hospital, Beijing, 100034 China

**Keywords:** Oncogene, Activatable nanoprobe, Molecular imaging, Message RNA

## Abstract

Malignant tumors have been one of the major reasons for deaths worldwide. Timely and accurate diagnosis as well as effective intervention of tumors play an essential role in the survival of patients. Genomic instability is the important foundation and feature of cancer, hence, in vivo oncogene imaging based on novel probes provides a valuable tool for the diagnosis of cancer at early-stage. However, the in vivo oncogene imaging is confronted with great challenge, due to the extremely low copies of oncogene in tumor cells. By combining with various novel activatable probes, the molecular imaging technologies provide a feasible approach to visualize oncogene in situ, and realize accurate treatment of tumor. This review aims to declare the design of nanoprobes responded to tumor associated DNA or RNA, and summarize their applications in detection and bioimaging for tumors. The significant challenges and prospective of oncogene-targeting nanoprobes towards tumors diagnosis are revealed as well.

## Introduction

According to the latest global cancer data reported by the International Agency for Research on Cancer (IARC) in 2020, there were about 19.29 million new cancer cases and about 9.96 million cancer deaths all over the world [1, 2]. In 2023, there are 1,958,310 new cancer cases and 609,820 deaths are projected to occur in the United States [3]. Cancer has become one of the major diseases that threatens people’s health and life. Cancer happens with the genetic mutation, leading to abnormal cells growing and proliferating unlimitedly and wildly with high changes in shape and in orientation soon [4]. The abnormal cells can invade surrounding tissues and shed into the blood or lymph circulation to expand to the whole body [5]. As a result, cancer cells expand their territory and damage the healthy tissues and organs leading to people’s death ultimately. Therefore, tumor research and clinical statistics show that timely diagnosis and effective intervention can significantly improve the therapeutic effect, greatly prolong the survival time, and largely alleviate the pain and mental economic burden of patients [6, 7]. However, most of cancers in the early phrase are largely asymptomatic, or just some nonspecific symptoms, including fever, nausea, vomiting, changes in stool characteristics, coughing, etc. which are often ignored [8].

At present, the early clinical diagnosis of tumors mainly depends on the biological detection and medical imaging [9–11]. The biological detection is composed by in vitro biological detection and tumor biopsy. The former including the immunological detection technology, gene chip and protein chip can provide some non-spatial-specific hints for occurrence and development of tumors, however it cannot accurately locate the tumors in the absence of imaging and pathology [12]. The latter requires invasive sampling of suspected tumor tissue, which is limited by the small sample size and obvious tumor heterogeneity. Additionally, the damage during sampling may also increase the risk of tumor metastasis [13].

In contrast with the biological detection, medical imaging can not only show the location of tumors from images directly, but also has the characteristics of non-invasion and safety [14–16]. Generally, the medical imaging technology for tumor diagnosis mainly contains computed tomography (CT), magnetic resonance imaging (MRI), positron emission tomography (PET), single photon emission computed tomography (SPECT), photoacoustic Imaging (PAI), and fluorescence imaging (FI) [17–19]. CT is a tomography image created by X-ray radiation source, usually jointly used with PET or SPECT, which detects the distribution or metabolic process of the labeled substance in vivo by radionuclides. PAI is the technology that detects ultrasonic signals generated by light excitation of tissue. FI is a common method that utilizes fluorescent labels which emit fluorescent signals after being excited. MRI is a biological magnetic spin imaging technology that utilizes the characteristics of nuclear spin motion. In an external magnetic field, a signal is generated after being excited by radio frequency pulses, obtaining high signal-noise ratio images.

With the aforementioned imaging techniques, the accuracy of tumor diagnosis has been greatly improved. In the past decade, the focus of medical imaging has been further transferred from the traditional imaging of tumor anatomical structure to the specific imaging of tumor malignant behavior related molecules or key targets, known as molecular imaging [20, 21]. In this context, different nanomaterials, especially those exhibiting unique intrinsic physical and chemical properties, have been proven to be powerful tools for biomedical applications. Through linking the imaging moieties for different imaging modalities and the targeting moieties for specific tumor hallmarks identification, a variety of nanoprobes have been constructed to realize the high-resolution molecular imaging. As more and more diverse probes with high sensitivity as well as specificity had been studied and constructed, molecular imaging technology has developed in a sustained and stable state in recent years. Consequently, there is no denying that molecular imaging probes occupy a significant position for tumor early diagnosis and early warning of metastasis [7, 16, 22].

Recently, owing to the capacity of controllable Watson-Crick base pairing, ease of synthesis and chemical modification, programmability, good biocompatibility and designability, nucleic acids probes are widely developed. DNA is emerged as a promising candidate more widely applied for biosensing, in vivo imaging and drug delivery in comparison with RNA, due to the inherent instability of RNA and the susceptibility to degradation by RNase [23–25]. So far, researchers have been constructed and reported well-designing oncogene-targeting nanoprobes with diverse nanostructures and nanomaterials using in the living animals for precise imaging of tumors.

**Fig. 1:**
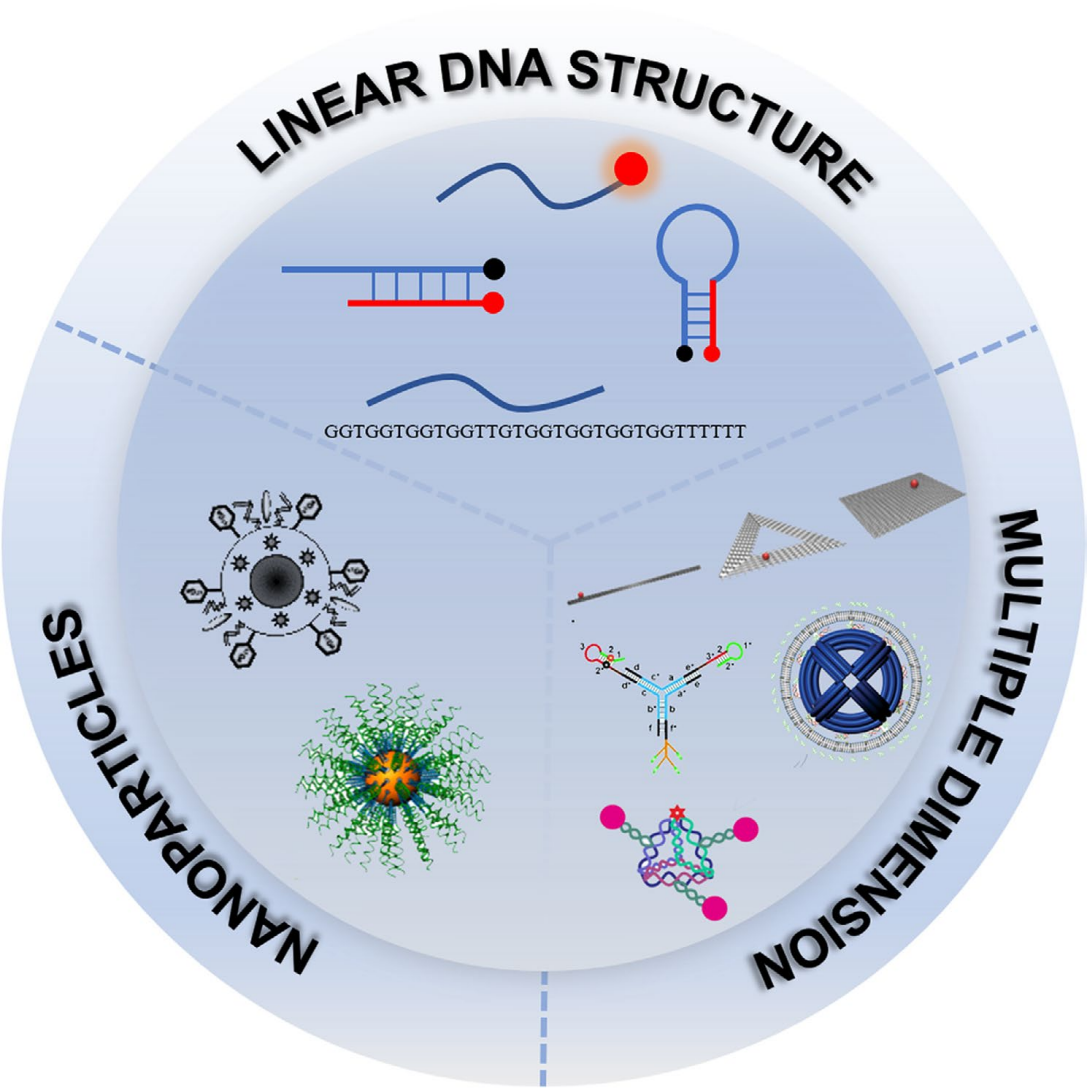
Schematic illustration of oncogene-targeting nanoprobes: construction and design

In the review, the well designs of oncogene-targeting nanoprobes with different nanostructures and nanomaterials were summarized (Fig. 1), and the state of art of their major applications for targeting and bioimaging in the living animals were discussed. In addition, the current challenges and future prospects of oncogene-targeting nanoprobes for both fundamental studies and potential clinical translation were presented.

## Design and development of oncogene-targeting nanoprobes

The earliest analysis techniques that utilized DNA are in situ hybridization and polymerase chain reaction (PCR), in which the cells were fixed and lysed [26, 27]. With the development of DNA technology, it is reported that linear DNA was the first sort of oncogene-targeting probes by using Watson-Crick hybridization principle for in vivo analysis, generally loaded with a single fluorophore applying for live intracellular analysis [28, 29]. However, the high background respond is difficult to be avoided with the single signal. To effectively address the problem, some researchers have designed oncogene-targeting probes based on linear DNA structure with multiple fluorophores with different fluorescent channels. Molecular beacons (MBs) are one of modified linear DNA designed with a hairpin structure that confines a fluorophore and quencher [30]. Because of the close proximity of the fluorophore and quencher, MBs exhibit no fluorescence signal. Once the probes seek their target molecules such as message RNAs (mRNAs), MBs will hybridize with the complementary sequences and emit a fluorescence signal because of the varying distance between fluorophore and quencher [31, 32]. Whereas, for lack of transfection reagents, some linear DNA probes can barely penetrate cell membranes and are degraded easily by nucleases in the cellular environment [24, 33, 34]. Additionally, owing to the no-target molecular on the probes, early DNA-based probes are mostly achieved by random diffusion of free DNA probes, with a relatively low efficiency and easy degradation before reaching the lesions [24]. In this context, the oncogene-targeting probes with linear structure and special sequences, such as DNA aptamers and DNAzymes, are designed and obtained artificially by in vitro selection Systematic Evolution of Ligands by Exponential Enrichment to recognize and target the specific molecules [35–37]. Specifically, DNA aptamers have the capability to recognize specific nucleic acids, proteins, ions, and other small molecules as well as pH, and for example, AS1411 aptamer molecules can specifically bind to overexpressed nucleolins on the surface of tumor cells [38–40]. DNAzymes can recognize and catalyze specific reactions such as cleavage of DNA or RNA, DNA modification, ligation and metalation of porphyrin rings [41–43]. Accordingly, the research and development of functional DNA have greatly provided much promise for improving the targeting and specificity of probes.

Owing to the inherent characteristics such as electric, optical, magnetic, mechanical, physiological and biocompatible, nanoparticles (NPs) have shown great potential for imaging probes, improving disease diagnosis and therapy. Some researchers have emerged NPs as the drugs delivery carriers because of the ability to deliver the cargo directly into cells or into desired areas in vivo [44–46]. Obviously, combining DNA with NPs can efficiently improve the stability and affinity of linear DNA. Some DNA designs with NPs could improve cellular uptake without transfection reagents [24, 47, 48]. The first designed nanoparticle-DNA complex was oligonucleotide-modified gold NPs (AuNPs), known as spherical nucleic acids (SNAs). Specifically, AuNPs were served as the core, which were functionalized with the nuclear acid strand. In addition, short fluorophore-labeled flare strands that can not only hybridize with nuclear acid on the surface of AuNPs, but also complement with target mRNA were introduced. After hybridizing on the NPs, the fluorescence of SNAs is quenched efficaciously due to the short distance between AuNPs and flare strands. However, when the recognition nucleic acid sequences in the flare strands bind with the targeting mRNA, the flare strand will be displaced, separating the fluorophore and AuNPs, thereby turning on the fluorescent signals. SNAs with three-dimensional orientation of nucleic acids possess numerous merits, including transfection-reagent-free uptake of cells, stronger binding affinity to their complements, minimal immunogenicity, and reduced propensity toward nuclease degradation using for regulating intracellular mRNA compared to corresponding linear DNA [47–49]. In addition to AuNPs, plasmonic NPs, metal NPs, carbon nanomaterials, and quantum dots (QDs) are also used for combination with DNA as well [34, 50–52].

Due to the great controllability and high precision of Watson-Crick base pairing of DNA, multi-dimensional DNA nanostructures have been designed to greatly improved performances of probes, which are capable of increasing biostability, enhancing cell internalization efficiency, accelerating reaction rate, and amplifying signal output [53, 54]. DNA nanostructures are consisted of one dimensional (1D), two dimensional (2D) and three dimensional (3D) structures ranging in different sizes or shapes such as tetrahedron, triangular prism, octahedron, icosahedron and complicated DNA origami structures [53, 55–57]. They have the ability of target accessibility and improve the sensitivity for various types of molecular targets (i.e., DNA, RNA, proteins, and other molecules) by several orders of magnitude [58, 59]. Nowadays, the above single or assembled oncogene-targeting strategy has been widely employed to design more sensitive, stable and biocompatible oncogene-targeting nanoprobes for in vivo analysis.

## The application for in vivo bioimaging

Detecting biomarkers of tumorigenesis such as accumulation of abnormal genes, changes in the tumor microenvironment (hypoxia, acidification, interstitial hypertension, vascular hyperpermeability, inflammatory reactivity, immunosuppression), etc., are one of the major approaches for designing the molecular imaging probes [22]. Cancer is caused by the accumulation of mutations in genes, thus, genetic instability is the basis and important feature of the occurrence and development of cancer. Malignant biological behaviors such as tumor occurrence, development, and metastasis is a complex pathophysiological process induced by multiple factors, involving multiple genes variations in different stages, which involves the activation of oncogenes, the inactivation of tumor suppressor genes and other related gene changes, resulting in cell proliferation, differentiation, and apoptosis [60, 61]. During the occurrence and development of tumors, DNA transcribe into mRNA or non-coding RNAs firstly, and then translate into related protein factors and receptors, etc. For example, microRNA (miRNA) is a non-coding endogenous small molecule RNA that participates in the post-transcriptional regulation of genes, which is closely related to tumorigenesis, metastasis, drug resistance and other pathological processes [62, 63]. Thus, RNA is directly related to the expression and regulation of genes, and the copy number of tumor-related RNAs is significantly increased compared with the number of cancer genes. According to the principle of Watson-Crick base pairing, oncogene-targeting nanoprobes can bind to tumor-related RNAs to obtain the accurate tumor in vivo imaging. Furthermore, abnormal proteins generated by tumor-related RNAs translation and proteins that participate and regulate related genes of tumors are also one of symbolic targets when cancers occur, such as RNA-binding protein (RBP) and nucleolin, etc. [64, 65] Tumor-related proteins are overexpressed in different cancer cell lines and endothelial cells, which the functional DNAs such as DNA aptamers and DNAzymes can recognize [66].

Molecular imaging probes constructed by targeting tumor-related biomarkers are expected to significantly improve the precision and specificity of tumors in vivo imaging, especially oncogene-targeting nanoprobes, which can identify mutated genes at an early stage of tumors for effective imaging. Simultaneously, with the nanomaterials modified or structure designed, oncogene-targeting nanoprobes can be ingested more efficiently by tumor cells. Therefore, on the basis of specific expression levels of tumor-related biomarkers, the stimuli responsive probes can be achieved, which is of great significance for promoting early cancer diagnosis, early warning of metastasis and accurate prognosis [67, 68].

### Linear DNA structure

**Fig. 2:**
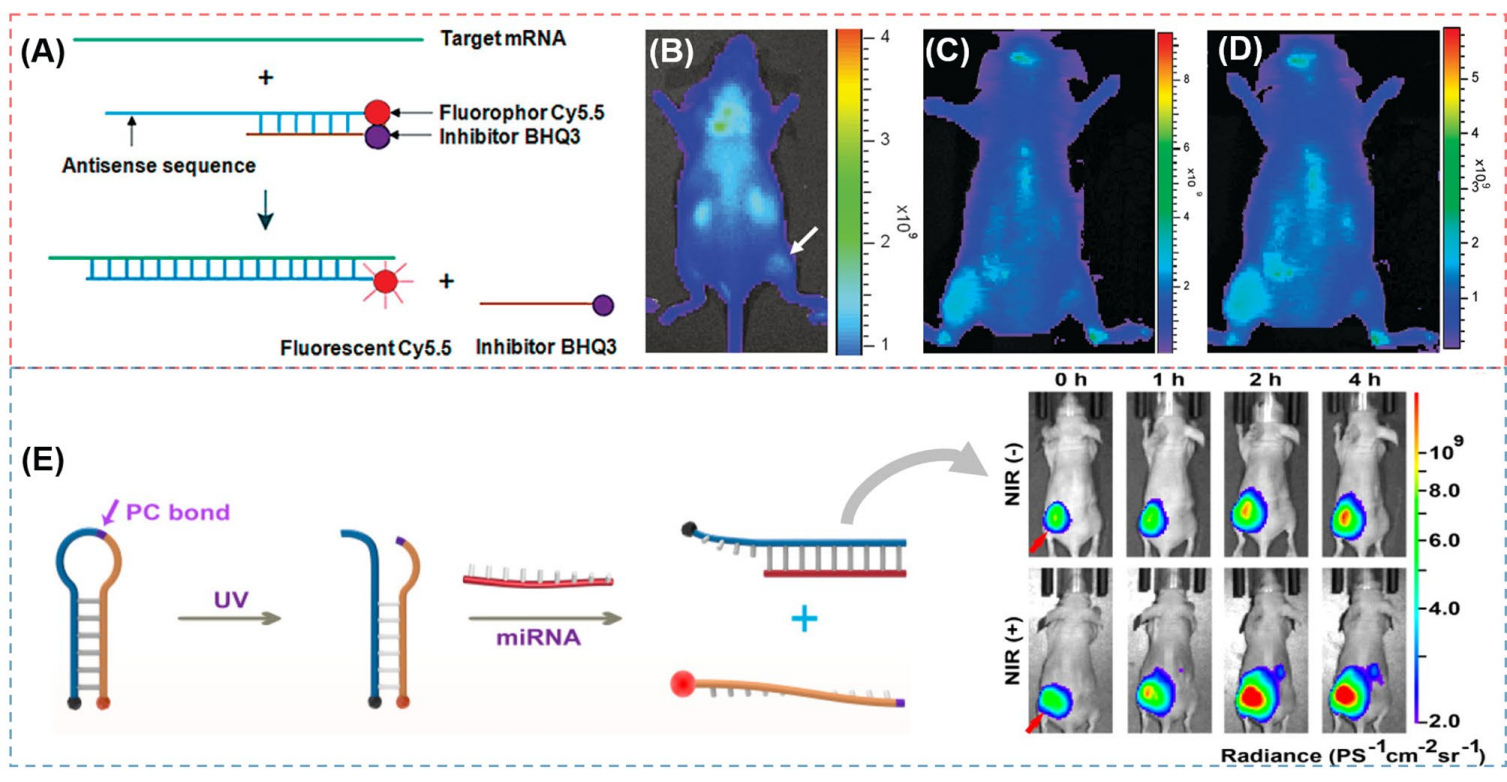
(**A**) Schematic diagram of DNA25-Cy5.5/cDNA-BHQ3 for KB-G2 tumor [70]; (**B**) Whole-body fluorescence images of KB-G2 tumor bearing mice at 5 h following administration of DNA25-Cy5.5/DNA18-BHQ3 duplex [69]; Whole-body fluorescence images of KB-G2 tumor bearing mice at 20 min (**C**) and 7 h (**D**) after injecting DNA25-Cy5.5/cDNA10-BHQ3 [70]; (**E**) Working principle of PBc-UN for NIR light and whole-body FI of HeLa tumor bearing mice after injection of PBc-UN with or without subsequent NIR illumination [71]. Reprinted with permission from ref [69–71]

Linear DNA is the single-stranded DNA that was the first sort of oncogene-targeting probes applied for in vivo analysis, which is widely combined with fluorescence and quencher to obtain the bioimaging with low background. Hnatowich and coworks [69] proposed a strategy of linear DNA (DNA25-Cy5.5/cDNA18-BHQ3) for KB-G2 (an epidermal carcinoma cell line) tumor in vivo imaging. A 25-mer phosphorothioate (PS) anti-mdr1 antisense DNA is conjugated with the Cy5.5 emitter on its 3’ equivalent end and hybridized as a linear duplex with a shorter 18-mer phosphodiester (PO) complementary DNA (cDNA) with the black hole inhibitor (BHQ3) on its 5’ end (Fig. 2A). Mdr1 mRNA (multidrug-resistant gene) was chosen as the target, which can be overexpressed in KB-G2 cells. Therefore, the authors injected DNA25-Cy5.5/cDNA18-BHQ3 into the KB-G2 tumor mice. After 30 min, the tumor/normal thigh fluorescence ratio was clearly positive, and reached a maximum at 5 h. It can be seen from Fig. 2B that an obvious accumulation of DNA probes can be observed in the tumor region. This is attributed that when the DNA probes closed to the target mRNA, they would form a new and stable antisense DNA25-Cy5.5/target mRNA duplex and release the shorter DNA along with its inhibitor. To further improve the fluorescence signal in tumor mice, in another work, Hnatowich and coworks [70] developed improved fluorescent DNA duplex probes with a 10 mer minor strand (PS DNA25-Cy5.5/PO cDNA10-BHQ3). The fluorescence signals are much higher than that of the 18 mer DNA25-Cy5.5/cDNA18-BHQ3 (Fig. 2C and D).

To better improve the precisely target performance, the development in the structure or the function of linear DNA has been studied such as MBs and DNA aptamers.

#### Molecular beacons (MBs)

Since the construction in 1996, MBs are in a common application for many biological fields such as genotyping, mutational analysis, the detection of PCR products and clinical diagnosis [30, 32]. MBs are DNA sequences composed of one target-recognition region of about 15–30 bases, which have a loop portion designed complementary to a desired target nucleic acid sequence, and a stem portion consisted of two self-complementary regions with five or six nucleotides [72]. A photoluminescent species (PLS) and a quencher at different ends of the stem with a close proximity result in a strong quenching of the photoluminescence. When hybridized with the target nuclear acid sequences, the MB loop portion changes into a linear structure, forcing the PLS and quencher far apart with the restoration of photoluminescence. Zhao et al. [71] developed a DNA nanodevice (PBc-UN) with MBs, functionalized upconversion NPs (UCNPs), and a photocleavable (PC) bond, which controls miRNA imaging by using NIR light. The MB is labeled with fluorophore Cy5 and black hole quencher (BHQ2) at the opposite ends respectively and the Cy5 is quenched because the close distance to BHQ2. When the NIR light penetrates deep tissues, a high-energy UV light is emitted by the UCNPs. Then, the photocleavable bond is broken and the MB can hybridize with the corresponding miRNA resulting in the activated fluorophore signal. Furthermore, the authors injected the probes into HeLa xenograft tumors on the left back of nude mice. It is observed in Fig. 2E that the PBc-UN + NIR treated group has a stronger fluorescence signal than the PBc-UN treated group. In another study, Sheng et al. [73] reported a human apurinic/apyrimidinic (AP) endonuclease 1 (APE1)-triggered MB probe (E-MBP/V), which is loaded on a PEGylated poly(aminoethylmethacrylate)-based tri-block co-polymer as a non-viral vector. The sensitivity and tissue specificity of MB are assessed by the amplification through identifying and targeting interleukin-6 (IL-6) mRNA. E-MBP embedded with two AP sites is labelled with a fluorophore and a quencher at the ends of the stem. When one E-MBP recognizes the targeting RNA, APE1 would cleave the beacon strand in the site of AP, while releasing the target RNA to react for the next cycle of identification and cleavage, and realizing signal amplification. In an acute paw inflammation model of Lipase (LPS) dealt of mice, the inflammation paws exhibited a sustained fluorescence enhancement with E-MBP/V, while no obvious fluorescence increased in PBS-treated paw. Compared with the MBP/V, a stronger fluorescence signal is observed in the E-MBP/V group due to APE1-mediated signal amplification. In this strategy, MBs are a promising way for intracellular mRNA monitoring and many other similar studies attached to macromolecules or nanoparticles can also applied for bioimaging [72, 74, 75].

#### Aptamer

Aptamers are single-stranded oligonucleotides that can recognize a wide range of potential biological targets, such as ions, small molecules, nucleic acids, peptides, and macromolecules, live cells and tissues sensitively [76]. Additionally, aptamers can penetrate tissue barriers and internalize in cells rapidly with high sensitivity due to their low molecular weight [77, 78]. However, the application of aptamers for in vivo cancer imaging remains limitation because of the poor stability in blood and the degradation by nucleases. Therefore, most of aptamers are utilized accompanied with the nuclear acid sequences, NPs or polymers, etc. [79–82] For example, Shi et al. [79] designed a novel locked nucleic acid (LNA)/DNA chimeric aptamer probe (TD05.6) with a DNA aptamer against lymphoma Ramos cells TD05 as the model through 7-base pair-LNA incorporation and 3’-3’-thymidine (3’-3’-T) capping supplement, which is used for tumor detection in serum and in vivo imaging. After intravenous injection of Cy5-labeled aptamers, Cy5-TD05 circulates within 30 min in the whole body including the tumor site with strong fluorescence signals and at 150 min Cy5-TD05 is cleared continuously with fluorescence signals fading. In contrast, Cy5-TD05.6 is at a much slower clearance rate both in target and nontarget areas due to the improved nuclease-resistant stability of LNA-modified aptamers. The signal of Cy5-TD05.6, even at 600 min, is still obvious in the tumor site. The effective imaging window is significantly extended from < 150 min of Cy5-TD05 to > 600 min of TD05.6, indicating that the combined LNA-substitution and 3’-3’-T-modification strategy could generate stable aptamer probes with substantially improved in vivo tumor imaging quality. In addition, Gong et al. [80] developed branched polyethyleneimine (PEI) protected aptamer molecular probes for specifically identifying tumors and efficiently increasing the circulation time. The probes utilize the PEI that can protect aptamers from DNase degradation and the aptamer TD05 that can target Ramos cells in Ramos tumor-bearing nude mice. Compared with the control DNA and the only TD05 aptamers, fluorescence signals of PEI/TD05 complexes from the tumor tissue are distinct at 30 min and remained increasing in 4–5 h after injection, which demonstrates the properties of targeting Ramos tumors and increasing the circulation time in mice.

**Fig. 3:**
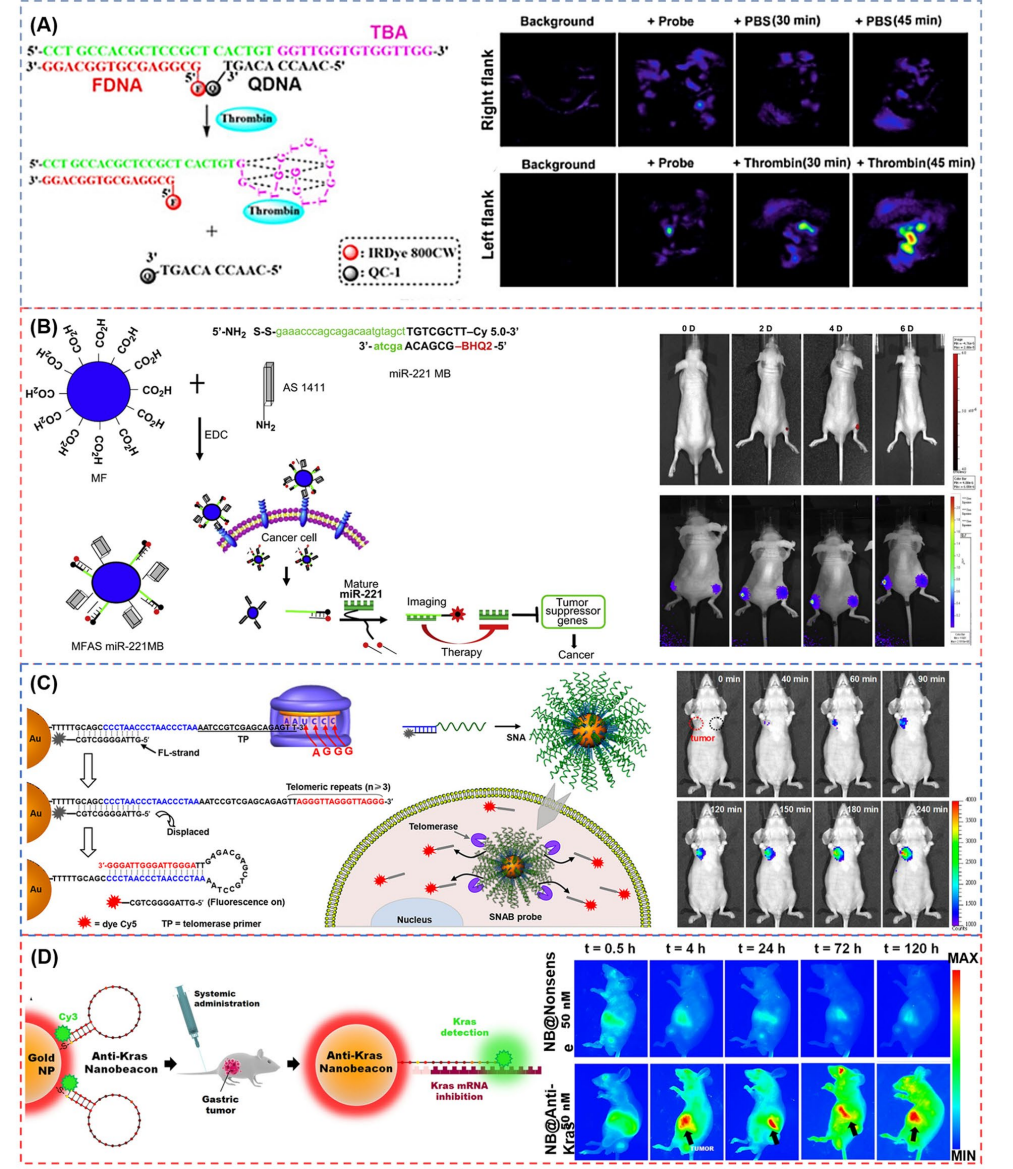
(**A**) Scheme of oncogene-targeting PA probe for ratiometric photoacoustic imaging of thrombin and in vivo PA imaging in response to PBS and thrombin [81]; (**B**) Strategy of cancer-targeting theragnostics using MFAS miR-221 MB and in vivo imaging of astrocytoma-targeting theragnostics with fluorescence imaging (upper panel) and bioluminescent imaging (low panel) [83]; (**C**) Schematic of the SNAB technology and telomerase detection in solution and time-lapse fluorescent images of tumor in vivo tumor (red circle) and tumor-free tissue (black circle) [74]; (**D**) Schematic diagram of AuNPs functionalized with a dye (Cy3) labeled hairpin-DNA and Epi-fluorescence analysis of nude mice bearing orthotopic human gastric cancer cells (0.5, 4, 24, 72 and 120 h) [75]. Reprinted with permission from ref [74, 75, 81, 83]

Aptamers are also frequently used as part of probes as targeting molecules, and are designed to an aggregation with better performance collocating with NPs or DNA structures. Zhang et al. [81] reported a functional DNA-based photoacoustic (PA) probe by using the DNA labeled with the fluorophore IRDye 800CW (FDNA) and the DNA labeled with the NIR dark quencher IRDye QC-1(QDNA), respectively. A thrombin binding aptamer (TBA) is selected as a recognition unit and thrombin as a target (Fig. 3A). When inactivated, TBA is closely bond with QDNA and the probe is in a quenching state due to the close proximity of two dyes. Nevertheless, when TBA combines with the thrombin, QDNA labeled with IRDye QC-1 is far away from main probes resulting in the luminescent signal. To test the feasibility of in vivo imaging by injecting the probes into the flanks of BALB/c mice, the thrombin and PBS (vehicle control) are injected to the left and right flanks, respectively. Only the site containing thrombin shows the signal observed in PA imaging in vivo. After the injection of 30 min, the PBS-treated mice show no significant difference of normalized PA780/PA725, and as the time increased from 30 to 45 min, the PA signal ratio increases, while the ratio increase at the thrombin-treated flanks is significantly higher.

To better recognize the disease site and make up for the insufficiency of a single probe, Kim et al. [83] designed a nanoparticle (MFAS miR-221 MB) by combining AS1411 aptamer and miRNA-221 MB (miR-221 MB)-conjugated magnetic fluorescence (MF) together to target, image and cure the cancer simultaneously (Fig. 3B). The AS1411 aptamer could target the nucleolin protein with high expression in cancer cells and the miR-221 MB could hybridize with the corresponding miRNA-221, inhibiting the expression of miRNA-221 to achieve the therapeutic effect. The FI (upper panel) and bioluminescent imaging (low panel) for 6 days are shown in Fig. 3B. The author implanted the astrocytoma with two kinds of C6 cells in the nude mice: one is the MFAS miR-221 MB and nuclear factor kappa B (NF-kB)/Firefly luciferase (Fluc) co-transfected into C6 cells in the right thigh, and the other is only NF-kB/Fluc in the left thigh as a control. It can be observed that the right thigh of MFAS miR-221 MB-treated mice are gradually visible at the fourth day and invisible at the sixth day after treatment, while the left thigh without MFAS miR-221 MB has no fluorescent signals (upper panel). In the bioluminescent imaging, MFAS miR-221 MB-treated mice have non-obvious signal for 6 days in the right thigh different from the left thigh with high bioluminescent signal gradually (lower panel). In another study, Shi et al. [82] constructed a hairpin-structured activatable aptamer probe (AAP) consisting of a specific sgc8 aptamer (A-strand), a poly-T linker (T-strand), and a short DNA sequence (C-strand) with a fluorophore and a quencher in order to detect the cell membrane protein tyrosine kinase-7 (PTK7) for human acute lymphoblastic leukemia CCRF-CEM cells. Once AAP contacts with the tumor site, it will bind with the protein and lead to the fluorophore activated. The fluorescence images demonstrate that AAP is activated with a dramatic fluorescence enhancement, and that AAP can be observed in the whole body rapidly at 15 min and almost be eliminated at 60 min, yet with the tumor site the most conspicuous. Comparing with the control probe and the “always-on” aptamer probe, AAP could be hardly influenced by the non-target tissues, which has a higher signal making the significantly enhanced image contrast as well as shortened detection time to 15 min.

### Nanoparticles (NPs)

Through conjugating biomolecules with nanomaterials, numerous novel hybrid materials applied in the medical field were generated. NPs with sizes in the range of 1-100 nm are demonstrated unique properties that these NPs are easily internalized into the solid tumors through the enhanced permeability and retention effect (EPR) [84, 85]. There exist a number of NPs-based therapeutics approving for clinical applications in solid tumors, such as Doxil (pegylated liposomal doxorubicin, ca. 100 nm) and Abraxane (albumin-bound paclitaxel NP, ca. 130 nm) [86, 87]. Up till now, the generally utilized nanomaterials for nanotechnology studies include organic and inorganic NPs, polymers, hydrogels, liposomes, polymeric micelles, and so on. In terms of organic and inorganic NPs, noble metal NPs, magnetic NPs, QDs, graphene or graphene-like two-dimension nanosheets, and magnetic NPs etc. have been studied [28].

AuNPs with the properties of ease in preparation and functionalized, biocompatibility, and size-dependent optical properties, which make them extremely attractive for sensors, imaging, drug delivery, therapy diagnostic and so on. In the past decades, AuNPs have been studied to conjugate with thiols, peptides, antibodies, and DNA molecules for the application of sensing, cell imaging, and drug delivery applications [88]. In this regard, it has been verified that both the stability and cell uptake efficiency of DNA are boosted after conjugating to AuNPs. Therefore, DNA-AuNP nanoconjugates become one of the most versatile hybrids bionanomaterials. At present, the development of DNA-AuNP fluorescent probes has been drawing increasing attention due to the strong fluorescence quenching of AuNPs. Furthermore, specific DNA sequences with multiple dimensions can increase the possibility of probes for imaging in vivo [89, 90]. For example, Liu et al. [74] designed a combination with spherical nucleic acids (SNAs) and exquisitely engineered MBs (spherical nucleic acid beacon, SNAB) to identify abnormal cells based on the molecular phenotype of telomerase activity. Telomerase is an enzyme responsible for the elongation of telomeres in cells, which is inhibited in normal human tissues but highly reactivated in tumors. Thus, the telomerase activity can be regarded as biomarker for tumor diagnosis [91–93]. The beacon has a long telomerase primer (TP)-carrying strand prehybridizing a short DNA strand modified by fluorophore (FL-strand) and is immobilized onto the AuNPs surface in a quenched state thanks to AuNPs. When associated to telomerase, the TP-carrying strand is recognized by the catalytic core of telomerase and changes the formation to stable DNA hairpin structure resulting in the separation of the FL-strand with fluorescence restoring. The authors established the xenograft cervical tumor models through planting HeLa cells into the subcutaneous armpit area of mice. As shown in Fig. 3C, SNAB can identify tumor cells and show the red fluorescence signal after 40 min injection compared with normal sites. As time passed by, the fluorescence signal gradually increased and covered the whole tumor mass confirming the SNAB effective activation. In another work, Conde’s group [94] developed a bio-responsive hydrogel-nanoprobe embedded with dark-gold NPs served as an activatable molecular nanoswitch loaded with the anticancer drug (5-fluorouracil (5-FU)), which can detect the triple-negative breast cancer cells (TNBC) and be triggered by hybridization with target multidrug resistance protein 1 (MRP1) mRNAs. The authors demonstrated that the bio-responsive hydrogel-nanoprobe could be anchored to the TNBC with in vivo imaging, simultaneously showing inhibitory effects on tumors, and only the nanobeacon anti-MRP1 with 5-FU has the ability to provide efficient inhibition on tumor progression, with an ∼90% tumor size reduction after 14 days. Bao et al. [75] proposed an in vivo universal tool consisted of functionalized gold NPs (AuNPs) with a Cy3 dye labeled MB to inhibit cancer cells development and metastasis in a murine tumor model. The MB can interlock and hybridize with target Kras mRNAs to inhibit the translation of the target protein and achieve the therapy effect. In vivo image shows the fluorescence emission over a period of 120 h (5 days) following nanobeacons injection (Fig. 3D). Only nanobeacons with anti-Kras are able to observe an obvious and efficient signal of the primary gastric tumor, and fluorescence signal is turned on at 4 h and maintained the signal until 120 h.

**Fig. 4:**
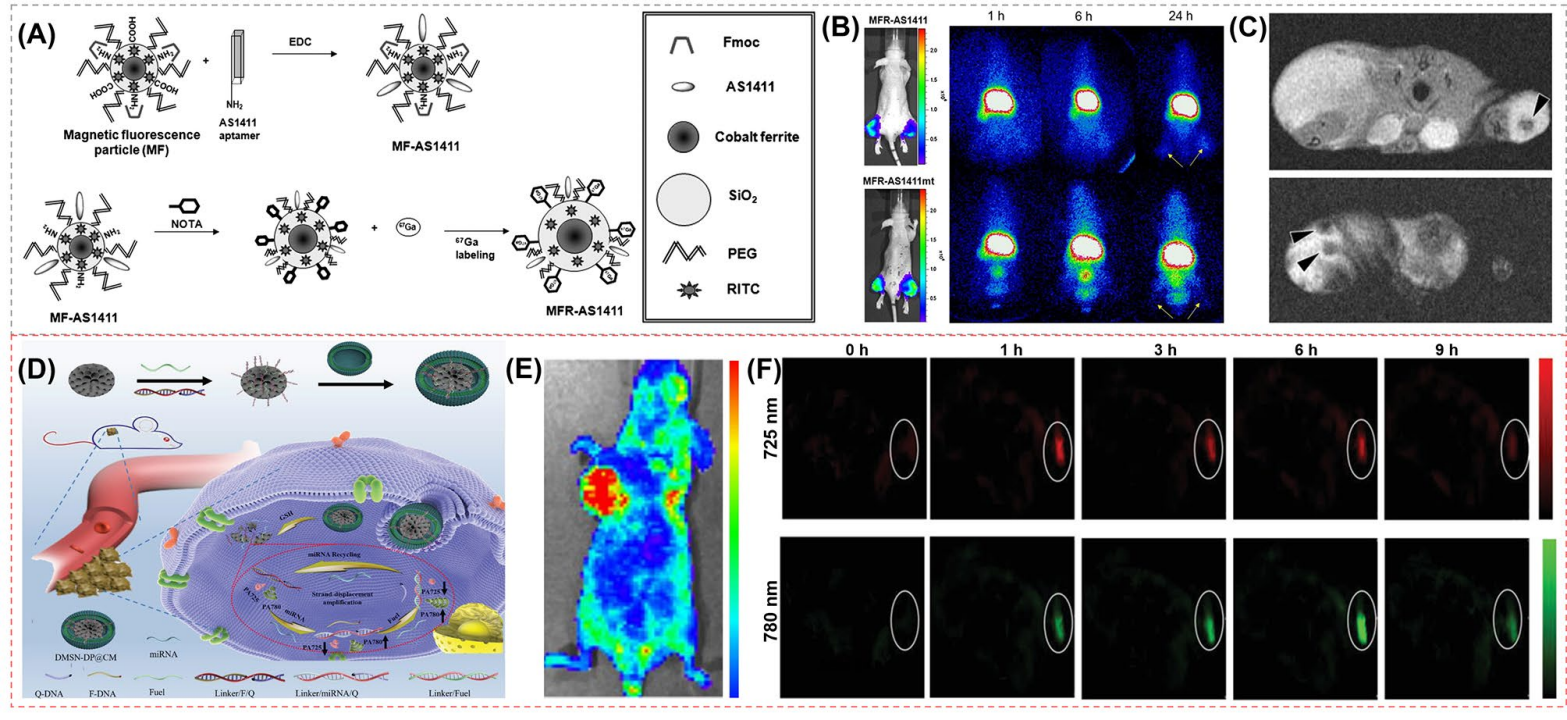
(**A**) Schematic illustration of steps involved in preparation of MFR-AS1411 [97]; (**B**) Radionuclide images of C6 tumors at 1, 6, and 24 h after injecting MFR-AS1411 and MFR-AS1411mt [97]; (**C**) MR images of tumor-bearing mice after injection of MFR-AS1411 [97]; (**D**) Schematic illustration of cancer cell membrane camouflaged nanoprobe (DMSN-DP@CM) for catalytic ratiometric PAI of miRNA detection in living mice [98]. (**E**) In vivo fluorescent images of MCF-7 tumor bearing mice were detected after injecting DMSN-DP@CM solution into the tail vein [98]. (**F**) PA images of MCF-7 tumor bearing mice treated with DMSN-DP@CM with different time points [98]. Reprinted with permission from ref [97, 98]

Apart from AuNPs, magnetic NPs (MNPs) attract substantial attention for cancer treatment as well due to excellent biocompatibility and degradation. Among MNPs, magnetite (Fe_3_O_4_), maghemite (Fe_2_O_3_) and iron oxide NPs (IONPs) are fully studied for biomedical applications [95]. Furthermore, cobalt-ferrite NPs (CoFe_2_O_4_ NPs) possess prominent physicochemical properties, mechanical hardness, improved stability and colloidal dispersibility under physiological conditions, more distinctive over other magnetic materials. Nowadays, CoFe_2_O_4_ NPs have been reported in nanobiosensing, bioseparation and purification, targeted drug delivery, imaging, etc. [96] Hwang et al. [97] utilized magnetic cobalt-ferrite NPs as the central core to construct a cancer-targeting multimodal imaging probe ^67^Ga-MNP@SiO2(RITC)-PEG/NH2-AS1411 (MFR-AS1411) coated with a silica shell and decorated by rhodamine B isothiocyanate fluorescence dye. Additionally, polyethylene glycol (PEG), Fmoc-protected amine moieties and a carboxyl group were surrounded with the surface of particles, which were further labeled with the AS1411 aptamer and p-SCN-bn-NOTA chelator (Fig. 4A). In bioimaging in vivo, the MFR-AS1411-administered mice show ^67^Ga radioactivity at the site of the administration, while the MFR-AS1411 mutant (MFR-AS1411 mt)-administered mice show rapid clearance via the bloodstream. The bioluminescence images (Fig. 4B) stably expressing luciferase in both thighs of MFR-AS1411- or MFR-AS1411mt-injected mice are clearly visualized, whereas the radionuclide signal is found only in both thighs of the MFR-AS1411-injected mouse. In MR images shown in Fig. 4C, *T*_2_-weighted MR images of tumor-bearing mice injected with MFR-AS1411 show the MFR-AS1411 particles as black spots. All these results indicate that the imaging probes can target nucleolin protein that highly expresses on the membrane of cancer cells, which can be monitored by fluorescent, radioisotope, and MRI modalities in vivo successfully and efficiently. In another study, Sun et al. [52] fabricated a theranostic MRI nanoprobe (PDA-DNA-DTPA/Gd nanoprobe) for imaging and treating tumors with the low toxic effect. The PDA-DNA-DTPA/Gd nanoprobe has a polydopamine (PDA) core coated by the single-stranded DNA S1 and its complementary strand DNA S2, and diethylenetriaminepentaacetic acid dianhydride (DTPA-DA) is bound to the ends of DNA S2 chelating Gd ions. This nanoprobe is inspired by the performance of DNA melting by the photothermal reagent, which can control the Gd releasing. Because of EPR effect, nanoprobes can potentially be enriched in tumor sites through passive targeting. After injecting the PDA-DNA-DTPA/Gd into the tumor site of the 4T1 tumor-bearing mice, the optimal contrast of the MRI signal is observed at 30 min. With the 808 nm laser irradiated, the *T*_1_ contrast of the non-irradiated tumor area has no change anymore, while the contrast of the tumor area following the laser irradiation weakens over time. After 1 h, the MRI signal is significantly lower than that without laser irradiation.

Owing to optical properties of strong luminescence, high photostability, and size-tunable emission wavelength, semiconductor QDs have received wide attention as a new generation of fluorescent probes [99, 100]. Similar with iron oxide nanocrystals, QDs with unique nanostructure are able to exhibit novel optical, electronic and magnetic properties. At a 5-100 nm diameter, QDs-based NPs, with large surface areas and functional groups, have the capability to link the multiple diagnostic modalities such as optical, radioisotopic or magnetic with cancer therapeutic approaches such as photothermal theray, photodynamic theray and immunotherapy [101]. Through assembling DNA with QDs, Dubertret et al. [102] encapsulated QD-micelles with phospholipid block-copolymer micelles and DNA to perform in vivo imaging. The authors selected the easily obtained Xenopus as the experiment target and microinjected in early-stage Xenopus embryos. The different stages and specific QD intracellular localizations can be observed by QD labeling in Xenopus embryos.

In addition, owing to the large radial pore structures and highly accessible surface areas, Dendritic mesoporous silica NP (DMSN) have attracted tremendous attention in biomedical applications as well [103]. For instance, Zhang et al. [98] designed cancer cell membrane camouflaged nanoprobe (DMSN-DP@CM) constructed by MCF-7 cell membrane-encapsulated DMSN. The nanoprobe is functionalized with DNA-photoacoustic (DNA-PA) probes and glutathione (GSH)-responsive DNA fuel strands (Fig. 4D). The near-infrared fluorophore/quencher pair (IRDye 800CW/IRDye QC-1) of DNA-PA probes can be activated by miRNA in living mice by trigger disassembly of multiple PA fluorophore probes from the quencher with the aid of GSH-responsive DNA fuel strands *via* the entropy-driven process. The PA signal was produced by two wavelengths (PA725 and PA780) corresponding to the absorption peak of the near-infrared fluorophore/quencher pair (IRDye 800CW/IRDye QC-1) on the DMSN-DP@CM, which can be recognized by target miRNA. When the probes are treated with the target miRNA-21, the contact-mediated quenching DNA behavior was relieved due to the miRNA-21-mediated displacement reaction, resulting in the ratio change of PA signals at the above two wavelengths. After injecting DMSN-DP@CM solution into the tail vein of the BALB/c mice with subcutaneous MCF-7 tumor, the fluorescence signal could be observed that DMSN-DP@CM was able to target and accumulate at the tumor site (Fig. 4E). As shown in Fig. 4F, the PA imaging shows that PA725 signal reached its maximum value after injection of 1 h and decreased along with time increasing, while PA780 signal exhibited reached its maximum value at 6 h.

**Fig. 5:**
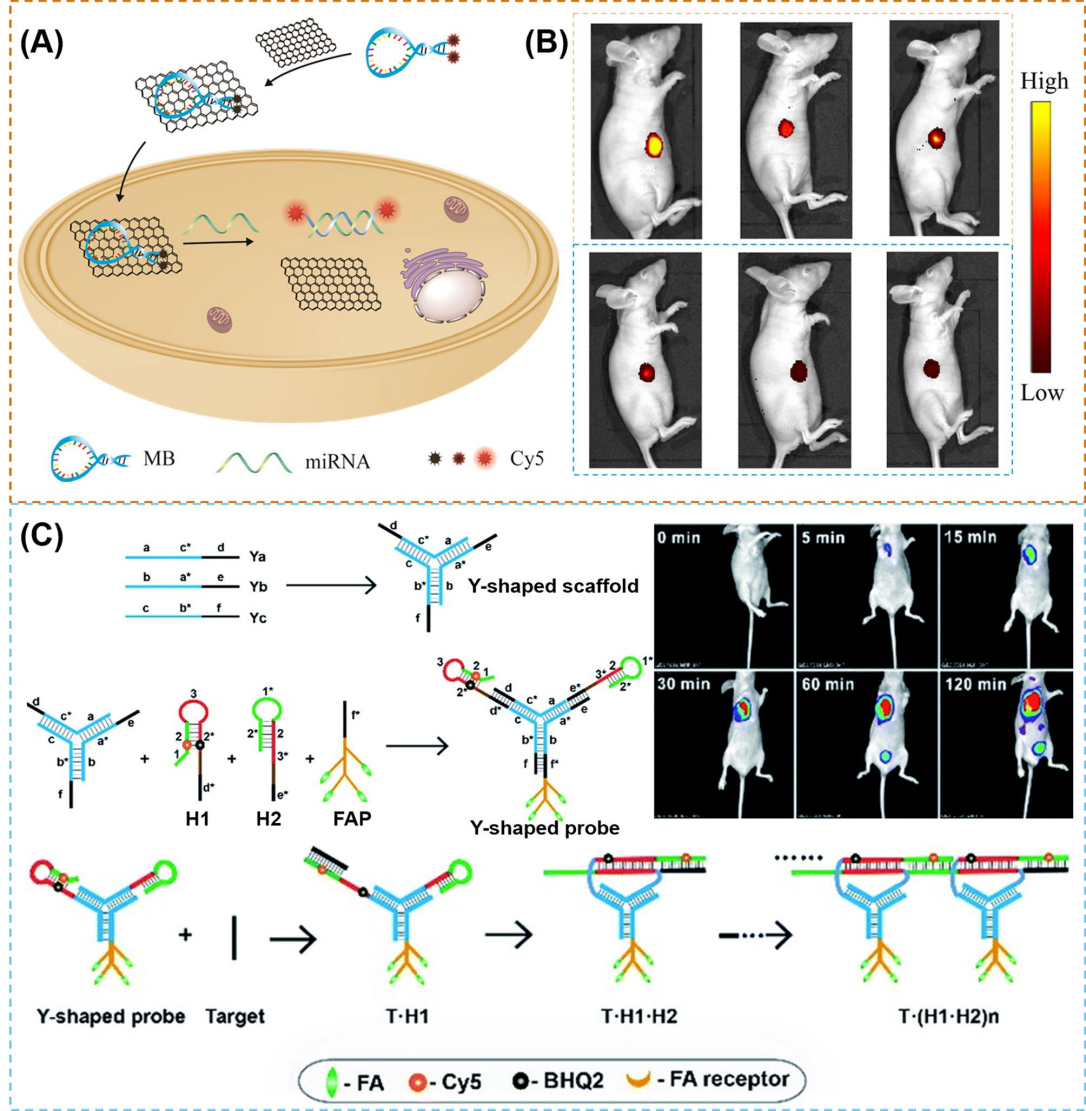
(**A**) Illustration of the formation of 2Cy5-MB-GO [107]; (**B**) In vivo imaging of three different xenograft tumor models (from left to right): MCF-7, HepG2, and HeLa. The upper is injected with 2Cy5-MB-GO, the bottom is injected with 1Cy5-MB-GO [107]; (**C**) Illustration of Y-H1-H2-FAP probe and time-dependent in vivo fluorescence images of HeLa tumor-bearing mice [108]. Reprinted with permission from ref [107, 108]

Graphene-based materials such as graphene oxide (GO) are a promising material to apply to photothermal treatment of cancer due to their wide NIR absorbance, large specific surface area and abundant functional groups [104]. Besides, graphene has π-rich conjugation, which can interact with single-stranded DNA (ssDNA) molecules *via* π-π stacking interactions. Beneficial from these properties, graphene can be a DNA carrier and a fluorescent quencher simultaneously [105, 106]. Yang et al. [107] reported a highly sensitive strategy that two Cy5 molecules were labeled onto the opposite ends of a single MB (2Cy5-MB) and the MB was adsorbed on the surface of GO (2Cy5-MB-GO) (Fig. 5A). Relying on the double-quenching effect, which is the graphene oxide (GO)-enhanced signal molecule quenching and the self-quenching effect of two Cy5, the fluorescence background was reduced. When meet the target microRNA-21 (miRNA-21), the 2Cy5-MB forms a duplex structure resulting in the 2Cy5-MB releasing from GO and two Cy5 molecules separating with the fluorescence signals restored. The fluorescence signal of 2Cy5-MB-GO can achieve about 156-fold increase in the presence of the miRNA-21 targets. As shown in Fig. 5B, the three xenograft tumor models with different tumor cell line (MCF-7, HeLa, and HepG2 cells) are used to assess the practicability of 2Cy5-MB-GO. As a results, 2Cy5-MB-GO can recognize the different tumor tissues with different fluorescence intensities and compared with 1Cy5-MB-GO, 2Cy5-MB-GO-treated tumor tissues display much higher fluorescence signals.

### Multi-dimensional DNA

Although nanomaterials create new opportunities for DNA probes on biological applications, cytotoxicity is still a severe problem, largely limiting their practical applications [24]. DNA itself is inherently non-cytotoxic as well-known, which could self-assemble to construct various multiple dimensional DNA probes, such as Y-shaped scaffolds, DNA tetrahedrons, polyhedrons, DNA dendrimers, and DNA origami. These multi-dimensional DNA structures are promising in biosensing, bioimaging, drug delivery, cell biology and material manufacturing applications [24, 109–112].

To greatly enhance resolution of tumor imaging, some researchers schemed out the amplified methods such as rolling circle amplification (RCA), strand displacement amplification (SDA), hybridization chain reaction (HCR), catalyzed hairpin assembly (CHA), etc. [113–115] For example, Wu et al. [67] utilized a signal amplification approach CHA designing a phosphorothioated tripartite DNA probe (Y-H1-H2-AS1411) that can show the FI of survivin mRNA in early-stage diagnosis of the sick mice. The DNA probe consists of three single-stranded DNAs, Ya, Yb and Yc *via* hybridization. Ya is linked with H2 (a hairpin probe decorated with a quencher moiety BHQ2), Yb is linked with AS1411 aptamer, and Yc is linked with H1(a hairpin probe decorated as well with a fluorescent reporter Cy5). As the AS1411 aptamer can mediate the probes to be ingested into the cells, once the tripartite DNA probes target and enter cancer cells, the target mRNA could hybridize with H1 and initiate the CHA cycle. Cy5 and BHQ2 of hairpin H2 are separated and the quenched fluorescence signal was activated subsequently. For RNA imaging in living mice, after being intravenously injected into a tumor-bearing mouse, the apparent signals can be observed at 0.5 h and the optimal intensity can be observed at 1 h, in which the DNA probe could mainly accumulate in the tumor region, liver and urinary bladder rapidly. After 8 h injection, the signal almost disappears. Furthermore, when the mice are injected with the non-targeting probe Y-H1-H2 and the non-amplification control probe Y-H6-H2-AS1411, only very weak fluorescence signals are observed in the tumor regions. In the same year, Wu et al. [108] utilized another signal amplification approach, hybridization chain reaction (HCR), developing an analogous phosphorothioated tripartite DNA probe (Y-H1-H2-FAP) that can hybrid with miRNA-21, which is overexpressing in the tumor. The DNA probe consists of three single-stranded DNAs, Ya, Yb and Yc *via* hybridization as well, and Ya is linked with H2 (a hairpin probe decorated with a quencher moiety BHQ2), Yb is linked with folate probe (FAP), and Yc is linked with H1(a hairpin probe decorated as well with a fluorescent reporter Cy5) (Fig. 5C). Because the folate (FA) receptors that overexpress in the tumor cells are recognized by FAP, the tripartite DNA probes could enter cells successfully and hybridize with the target mRNA with H1, and initiate the CHA cycle, following by the activation of quenched fluorescence signal. In the in vivo RNA imaging of mice, the signal exhibits a rapid increase in the fluorescence responses in the HeLa tumors at 2 h post-injection.

DNA tetrahedron has been considered as one of the most practical DNA nanocage structures due to its simply assembled capacity from four DNA strands and preparation in high yield. Furthermore, the tetrahedron is significantly resistant to nucleases, which makes the DNA tetrahedron ideal for in vivo imaging [24, 110]. Lee et al. [116] utilized the Watson-Crick base complementation to self-assemble a tetrahedron oligonucleotide NPs (ONPs) with FA and Cy5. It is proved that ONPs are capable of targeting the lesion of KB tumors, and simultaneously the CT scanning image and the three-dimensional FMT-CT image reveal the apparent tumor site of a tumor-bearing mouse. Compared to the simple structure probe (FA-conjugated siRNA) with the self-assembled probe (ONPs) through the in vivo live fluorescence images, ONPs have a stronger signal with the same measurement of FA-conjugated siRNA.

**Fig. 6:**
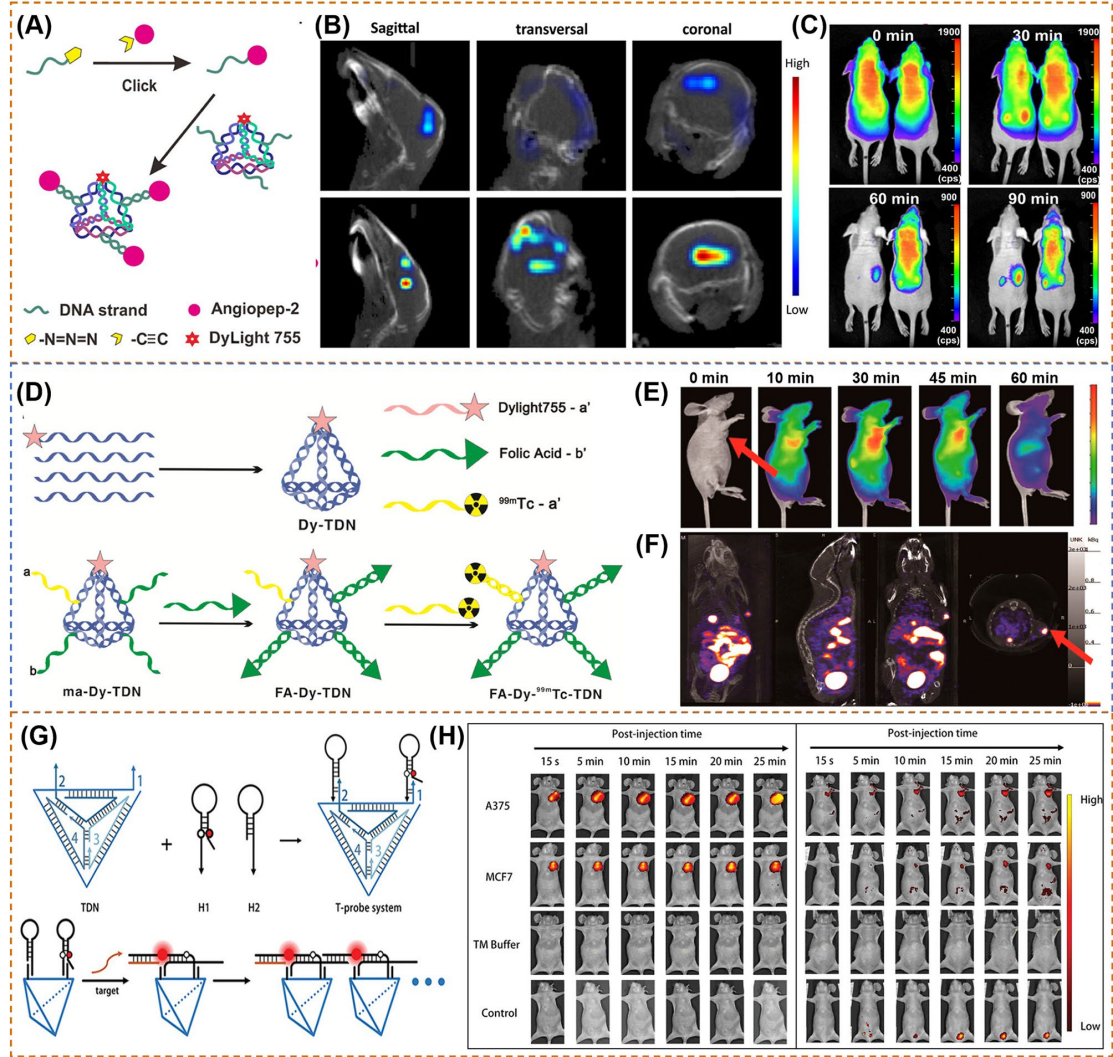
(**A**) Synthesis scheme of DNA tetrahedron structure of ANG-TDNs (**A**) [117]; (**B**) 3D reconstruction of in vivo imaging of TDNs (top) or ANG-TDNs (bottom) treated mice [117]; (**C**) In vivo fluorescent images after injecting TDNs (left) or ANG-TDNs (right) for 90 min in normal mice [117]; (**D**) Synthesis scheme of DNA tetrahedron structure of FA-Dy-99mTc-TDN [118]; (**E**) Dynamic biodistribution of monitored in the KB tumor-bearing nude mice for continuous 1 h through fluorescent imaging [118]; (**F**) SPECT-CT imaging of FA-Dy-99mTc-TDN in the KB tumor-bearing nude mice 2 h post injection [118]; (**G**) Schematic for the fabrication of the T-probe system and amplified imaging of multiplexed target with T-probe *via* hybridization chain reaction (HCR) [119]; (**H**) In vivo fluorescence images obtained in 25 min after peripheral tumor injection (left) and after tail-vein injection (right) [119]. Reprinted with permission from ref [117–119]

Tian et al. [117] utilized the tetrahedral DNA nanostructures (TDNs) with the property of internalization in living cells as well as high resistance to degradation to develop a biocompatible framework nucleic acid (FNA)-based targeting probe for brain tumor imaging (Fig. 6A). The probes (ANG-TDNs) are modified with angiopep-2 (ANG) and NIR dyes. In vivo imaging studies (Fig. 6B) in mice show that the fluorescent signal of ANG-TDNs is accumulated in the brain area, indicating the more efficient BBB shuttling of ANG-TDNs. Furthermore, after 90 min injection in glioma mice (Fig. 6C), the fluorescence intensity of ANG-TDNs is mainly focused on the brain tumor region compared with TDNs, indicating that ANG-TDNs could cross the blood − brain barrier (BBB) and target the glioma site. Jiang et al. [118] developed a multiple-armed tetrahedral DNA nanostructures (TDNs) by combining fluorescent dye (Dylight 755), FA-conjugated ssDNA and radioactive isotope technetium-99 m (^99m^Tc)-labeled ssDNA together (Fig. 6D). After intravenous tail injection of KB tumor-bearing mice (Fig. 6E), the authors found that FA-Dy-99mTc-TDN can accumulate at the area of the tumor effectively comparing to the no FA probes and free FA probes. The NIR and SPECT imaging show the similar phenomenon that the signals are mainly at the tumor site, liver, and bladder (Fig. 6F). In another work, Kim et al. [101] fabricated a tetrahedral DNA nanoparticle (Cy5-Td) with Cy5 labeled for detecting sentinel lymph nodes (SLN). Comparing with Cy5-Td, the linear duplex DNA with Cy5 (Cy5-Ds) shows lower fluorescence signal and fails to provide clear spatial information. Thus, tetrahedral DNA structure is a promising nanomaterial for in vivo imaging with the lower background signals. Shedding new light on different DNA structure technology, Zhang et al. [119] designed a complicated tetrahedral framework DNA-enhanced (TDN-enhanced) HCR detection system (T-probe system) for cancer-related detection. As shown in Fig. 6G, the T-probe system consists of a TDN and two hairpin probes (H1 and H2) with fluorescein and quencher labeled in the stem region of H1. When detecting the target, H1 could open *via* a toehold-mediated strand displacement (TMSD) reaction, and H2 opens as well with the hybridization of H1, triggering the further HCR and the activatable fluorescence signal, resulting in further fluorescence signal amplification. After peripheral-tumor injection and tail vein administration, the fluorescence signal in the A375/MCF7 tumor can be observed (Fig. 6H). Obviously, a rapid response of probes can be instantly observed at the region of the tumor, and the fluorescence intensity remains high level at 25 min compared with the control hairpins and TM buffer. In addition, the probes accurate targeting capabilities with an obvious fluorescence signal appeared at 10 min in A375 tumor and 20 min in MCF7 tumor after intravenous injection of probes.

**Fig. 7:**
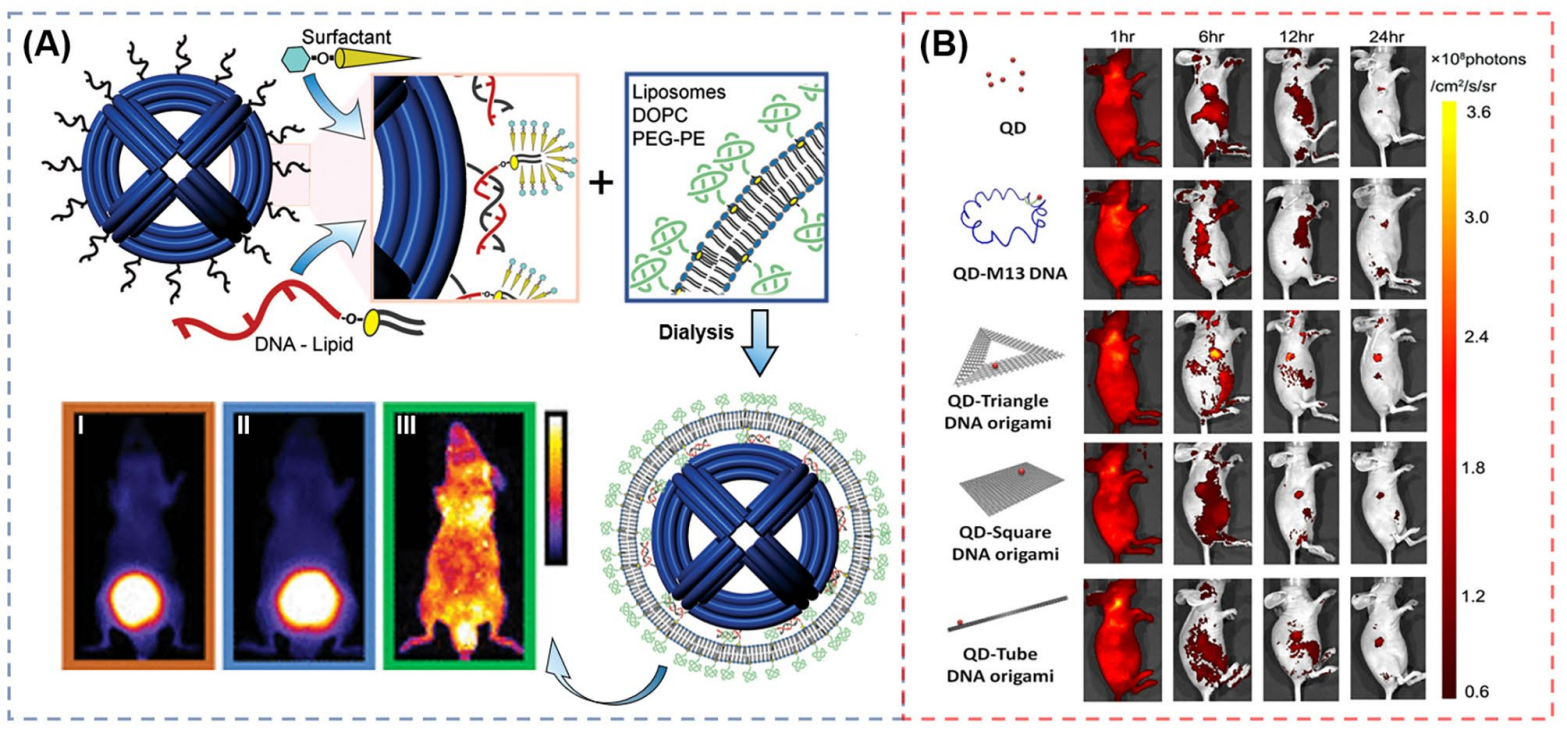
(**A**) Schematic of the encapsulation strategy and in vivo optical imaging for analysis of pharmacokinetics and biodistribution. (**B**) Dynamic biodistribution of QD, QD-M13 DNA, QD-triangle DNA origami, QD-tube DNA origami, and QD-square DNA origami monitored in the tumor-bearing nude mice for continuous 24 h through FI Reprinted with permission from ref [120, 121]

Polyhedrons have excellent advantages over tetrahedrons such as more recognition chains with an increasing reaction rate, plenty of polygon edges with more flexible design and greater utilization rate of the polygonal cavity, leading to broad application in bioimaging and drug delivery therapy [24]. Shih et al. [120] constructed a wireframe DNA nano-octahedron (DNO) with a lipid-bilayer. As shown in Fig. 7A, lipid-DNA conjugating as staple strands is firstly self-assembled with bacteriophage-derived scaffold DNA as the template strand in a surfactant solution, following with annealing to the outer handles of non-encapsulated DNA nano-octahedron (N-DNO), which form micelles around the conjugates. Then, Liposomes are added and the mixed surfactant-lipid micelles are obtained. Finally, Dialysis selectively removes the surfactant and results in a fused lipid bilayer around the DNO, forming the encapsulated DNO (E-DNO). In vivo optical imaging shows that after injecting 120 min, E-DNO distribute throughout the body, while the controlled AlexaFluor750-labeled oligonucleotide and N-DNO accumulated in the bladder.

DNA origami has received much attention as well in the areas of cellular imaging, targeted payload delivery, and controlled drug release, which can be folded into arbitrary shapes through a long single strand of DNA hybridizing with hundreds of short DNA strands to construct a special functional DNA structure [56, 57]. DNA origami can be chemically modified and integrated with various biomolecules and NPs [56, 121]. For instance, Zhang et al. [121] assembled three kinds of DNA origami nanostructures including triangular DNA, squared DNA, and tube-shaped DNA conjugated with QDs for tumor therapy in MDA-MB-231 tumor-bearing mice. As seen in the FI (Fig. 7B), triangle DNA exhibits the best accumulation (309.3 ± 33.1 × 10^6^ p/s/cm^2^/sr) than the squared DNA (117.7 ± 5.0 × 10^6^ p/s/cm^2^/sr) and tube-shaped DNA (124.3 ± 10.3 × 10^6^p/s/cm^2^/sr) due to the most desired passive tumor targeting ability. In addition, triangular DNA possesses the ability of long-term tumor uptake in vivo after observing the ex vivo FI of the tumor. The triangle DNA is mainly accumulated in tumor during 24 h. After verify the tumor passive targeting and long-lasting properties at the tumor region of the DNA origami nanostructures, the author loaded the anti-cancer drug doxorubicin into the DNA origami, which exhibits a significant antitumor efficacy for breast-tumor-bearing mice without obvious toxicity.

To improve the detection sensitivity and target recognition specificity of DNA nanoprobes, multiple strategies are integrated together [122–124]. For example, Yang et al. [124] assembled a multivalent DNA triangular prism (DTP) through a streptavidin linking three biotinylated DTPs. CHA reactions are introduced to realized sensitive, rapid and multiplexed imaging of miRNA in living cells.

## Summary and outlook

Molecular imaging technology occupies a significant position for tumor early diagnosis and timely warning of metastasis, which can detect variation of diseases at a molecular level with non-invasive ways. Playing a vital role in molecular imaging development, probes determine the development and improvement direction of medical imaging technology. Up till now, researchers have been continuously designing and fabricating a variety of probes for imaging cancer biomarkers in vivo with high sensitivity and accuracy. Oncogene-targeting nanoprobes has been emerged as a promising candidate to detect the early tumor site and deliver drugs due to the capacity of controllable Watson-Crick base pairing, ease of synthesis and chemical modification, programmability, good biocompatibility and designability.

In this review, we have summarized the designs and constructions of several typical oncogene-targeting probes for in vivo imaging in living animals, and enumerate examples about the applications in different molecular imaging technology. Linear DNA probes was reported to be the first probe utilized in vivo, frequently with fluorophore now, following with the occurrence of MB increasing the accuracy as well as decreasing the background signal and functional DNA sequences (e.g., DNA aptamers, DNAzyme) improving the specific recognition. However, single-stranded nucleic acid barely shows efficient cell internalization due to their small size and negatively charged backbone, and show easy degradation by nucleases before reaching the targets. Up to now, with the DNA nanotechnology development, oncogene-targeting nanoprobes successfully evolve assembled with NPs and diverse structures. NPs such as AuNPs, SiO_2_, graphene, QDs, magnetic NPs and so on have been used to adjust the pharmacokinetics of loaded DNA molecules. Transformation of structure such as tetrahedron, triangular prism, octahedron, icosahedron and complicated DNA origami provide more design options with biocompatibility and designability. In terms of especial and excellent oncogene-targeting nanoprobes designed and constructed, the target accessibility and the sensitivity for various types of molecular targets (DNA, RNA, proteins, and small molecules) are enhanced by several orders of magnitude.

However, the development of oncogene-targeting nanoprobes still exist limitation by several challenges. Long-term biological toxicity is emerged as the vital concerned issue. Oncogene-targeting nanoprobes, especially labelled with NPs, exist potential risk when they are utilized in the process of the phagocytosis, opsonization, and endocytosis of normal cells. It is reported that some NPs may cause the potential adverse side effects of the liver, spleen, kidneys, lymph nodes, heart, lungs, and bone marrow [125, 126]. Therefore, it is imperative to develop and improve highly biocompatible nanomaterials with low toxicity for clinic applications. What’s more, there is doubt whether DNA nanostructures designed artificially will cause genetic diseases.

The cost and feasibility of synthesis of DNA are brought to the forefront of attention as well. For one example, it is difficult and expensive to synthesize such long DNA sequences (> 100 nt). For the other example, DNA origami structures need hundreds of DNA strands to construct increasing the total costs. Additionally, the large-scale production and purity of multiplied dimensional DNA nanostructures still need to be improved. Moreover, their complicated structures demand high concentrations of Mg^2+^ to maintain structural integrity avoiding the degradation of enzymatic hydrolysis, which is a high level for cellular microenvironment.

The controllability of oncogene-targeting nanoprobes is still a focus when they are delivered to the tumor sites. Many reported DNA probes for in vivo imaging exist insufficient time-space sensitivity and thus may turn on their signals during the delivery process, leading to the inaccuracy of results. Furthermore, the low concentration of biomarkers limits the probe sensitivity and selectivity and more amplification techniques are used for imaging such as rolling circle amplification (RCA), strand displacement amplification (SDA), hybridization chain reaction (HCR), catalyzed hairpin assembly (CHA) and so on. It is requisite to explore more suitable methods to successfully amplify biomarkers [111, 113].

In the prospect, the development of oncogene-targeting nanoprobes has infinite possibilities. With specific advantages of various DNA nanoprobes, for example, nanoprobes with NPs are better for controlled drug delivery and release, and DNA self-assembly structures have better biocompatibility, more and more multi-modes and multi-functional oncogene-targeting nanoprobes with novel structures and different materials will be developed, which can provide more detailed and sensitive information about diseased tissue in vivo. Besides as probes for in vivo imaging or as drug delivery carriers, oncogene-targeting nanomaterials can be deeply integrated and applied to different fields in smart drug delivery vehicles, nanorobots, biosensors, energy storage, and materials science. With the innovative design with combination and structures as well as the intersection of multiple disciplines, these potential nanotechnologies will have broad application prospects towards the direction of multi-functional and multi-modal.

## Data Availability

The data used to support this review are included within the article.
